# Gaussian Process Autoregression for Joint Angle Prediction Based on sEMG Signals

**DOI:** 10.3389/fpubh.2021.685596

**Published:** 2021-05-21

**Authors:** Jie Liang, Zhengyi Shi, Feifei Zhu, Wenxin Chen, Xin Chen, Yurong Li

**Affiliations:** ^1^Department of Rehabilitation, Fuzhou Second Hospital Affiliated to Xiamen University, Fuzhou, China; ^2^College of Electrical Engineering and Automation, Fuzhou University, Fuzhou, China; ^3^Fujian Key Laboratory of Medical Instrumentation and Pharmaceutical Technology, Fuzhou, China

**Keywords:** sEMG, Gaussian process, joint angle prediction, NARX, neurorehabilitation

## Abstract

There is uncertainty in the neuromusculoskeletal system, and deterministic models cannot describe this significant presence of uncertainty, affecting the accuracy of model predictions. In this paper, a knee joint angle prediction model based on surface electromyography (sEMG) signals is proposed. To address the instability of EMG signals and the uncertainty of the neuromusculoskeletal system, a non-parametric probabilistic model is developed using a Gaussian process model combined with the physiological properties of muscle activation. Since the neuromusculoskeletal system is a dynamic system, the Gaussian process model is further combined with a non-linear autoregressive with eXogenous inputs (NARX) model to create a Gaussian process autoregression model. In this paper, the normalized root mean square error (NRMSE) and the correlation coefficient (CC) are compared between the joint angle prediction results of the Gaussian process autoregressive model prediction and the actual joint angle under three test scenarios: speed-dependent, multi-speed and speed-independent. The mean of NRMSE and the mean of CC for all test scenarios in the healthy subjects dataset and the hemiplegic patients dataset outperform the results of the Gaussian process model, with significant differences (*p* < 0.05 and *p* < 0.05, *p* < 0.05 and *p* < 0.05). From the perspective of uncertainty, a non-parametric probabilistic model for joint angle prediction is established by using Gaussian process autoregressive model to achieve accurate prediction of human movement.

## 1. Introduction

Rehabilitation robots and other rehabilitation equipment have developed rapidly and are widely used for therapeutic training of patients suffering from neurological disorders including stroke, cerebral palsy and spinal cord injury. Patients with impaired neurological function are able to use rehabilitation equipment for a variety of exercises to restore strength and flexibility in their extremities (1). Neurorehabilitation techniques can help both passive and active training of patients with neurological injuries. Compared to passive training through rehabilitation equipment, training that involves the patient's own will can improve the effectiveness of treatment and restore motor function through active movement (2). Electromyography (EMG) represents the sum of subcutaneous motor action potentials generated through muscle contraction (3), which can represent neuromuscular activity and is a way to reflect the patient's voluntary effort. Surface electromyography (sEMG) is a non-invasive EMG signal that is mainly acquired through non-invasive electrodes, which has the advantage of being more accessible and less likely to impede the normal activity of the user and will override the actual joint motion (4), and is often used as a control command to realize the human-robot interface (HRI) of portable or wearable assisted rehabilitation equipment. The surface electromyography (sEMG) is a non-invasive EMG signal collected by surface electrodes, which has the advantage of being more accessible than electroencephalography (EEG) and less likely to impede the user's normal activities. The forces generated by muscles in response to neural control signals depend on a large number of variables distributed over many spatiotemporal scales (5), which makes it difficult to predict the muscle force. While the nervous system clearly has knowledge of some of these variables, such as muscle length and velocity, other aspects of muscle dynamics (e.g., the detailed dynamics of molecules at the level of individual myofibrils and sarcomeres) are much more difficult to measure and estimate. With knowledge of the nervous system, it is possible to model the relationship between neural control signals and muscle force and use it to predict or simulate muscle force production. However, this relationship itself cannot be accurately estimated, and the remaining unaccounted aspects of muscle dynamics will result in seemingly random fluctuations in force, also known as motor noise. Therefore, there are two main sources of uncertainty in the neuromusculoskeletal system: irreducible noise during the motor system and variability in the relationship between neural control signals and muscle outputs (6). The impact of uncertainty in neuromusculoskeletal models on joint motion prediction can be mitigated, but not completely eliminated, by different modeling approaches. The human neuromuscular system is a highly non-linear and time-varying system with uncertainty (7). In order to allow models to handle the dynamic high-dimensional nature of the neuromuscular system, it is not enough to rely on traditional model structures and faster computational processing. Therefore, a central issue for further research on neuromusculoskeletal systems, or any artificial controller, is how to command muscles effectively in the presence of uncertainty.

Non-linear system modeling and identification can be divided into parametric and non-parametric models from the perspective of model structure (8–12). From the perspective of the Bayesian statistical framework, probability distributions over the functional space can be considered and modeled by optimizing these distributions to characterize uncertainty. This type of model has no explicit modeling mechanism or constraints and is referred as non-parametric modeling (13). The non-parametric model does not depend on a specified set of parameters or a fixed model structure, and is an estimation method based on statistical principles, which can generate functions to fit the data without the constraints of the model mechanism. The number and nature of the parameters of the non-parametric model are flexible and variable, which can change accordingly with the change of the data set. It can be used for modeling and analyzing high-dimensional time-varying systems, taking the uncertainty into account during the modeling process, and characterizing the uncertainty. The non-parametric methods mainly include spectral estimation, spectral analysis, correlation analysis and kernel-based analysis methods. Gaussian process (GP) model is a non-parametric kernel method in the framework of Bayesian model, which is simple to implement, computationally efficient, and most importantly, GP model can describe the posterior distribution of the model function, which in turn can be used to describe the uncertainty of the model, and is a recent research hotspot for non-parametric methods (14, 15). Kang et al. proposed an effective method for generating suboptimal motion of a multi-body system using a GP dynamics model to achieve dimensionality reduction of the system and deal with motion optimization problems (16). Schearer and Ullauri achieved the estimation of joint moments by building semi-parametric and non-parametric models through GP model, respectively (17, 18). Xiloyannis et al. used a Gaussian autoregressive model for decoding neural information to achieve multidimensional decoding control of 11 joint movements (19). Yang et al. proposed a proportional pattern recognition control of arm muscles using a wearable ultrasound sensor to achieve both gesture recognition and muscle contraction force estimation based on statistical features and Gaussian process regression models (20).

It has been shown that joint motion can reflect the intrinsic dynamics of human movement (21), so motion signals can also be used in the modeling of the neuromusculoskeletal system for building autoregressive models for prediction. The non-linear autoregressive with eXogenous inputs (NARX) model is an effective method for solving non-linear sequential problems, and modeling in conjunction with the NARX model can better incorporate the non-linear spatiotemporal correlation structure of muscle-driven control signals and natural human motion (22). Dynamic recurrent neural networks based on the NARX model are widely used for joint angle estimation, decoding shoulder, elbow and wrist motions and prosthesis model control (23–25). Gupta proposed an ankle joint angle estimation model based on the NARX model using sEMG signals and knee joint angle signals, and the performance of the model proved its applicability to ankle joint angle estimation for active prosthesis, orthosis and lower limb rehabilitation (26). Liu et al. used the NARX model to train and identify the EMG signals motion mapping relationship between a rehabilitation training bed and sEMG signals based motion prediction to achieve the identification of upper body tilt in different directions (27). Raj et al. proposed a multilayer perceptron neural network model based on the NARX model for estimating elbow joint angle and elbow joint angular velocity, and the proposed model estimated elbow joint angle and elbow joint angular velocity with high accuracy (28).

Since the neuromusculoskeletal system is a dynamic time-varying system, the GP model is only a mapping of the input to the output distribution, which is a basic static system. By combining the NARX model into the modeling, the resulting model can not only adapt to non-linear discrete-time processes, but also to different noise models, and the resulting dynamic model, which will better fit the physiological properties of the neuromusculoskeletal system. Thus, in this paper, a knee joint angle prediction model based on sEMG signals is proposed by considering the physiological properties of microscopic muscle activation and combining NARX with GP model. First, considering the physiological properties of sEMG, the muscle activation kinetic model is used to extract features from the sEMG signals of a pair of antagonist muscles controlling knee joint motion, and the muscle activation intensity of this pair is obtained. Then a joint angle prediction model based on GP model is proposed for the uncertainty of the neuromusculoskeletal system with the muscle activation intensity as the input signal. Since the neuromusculoskeletal system is a time-varying non-linear system, the one-step ahead (OSA) prediction of the NARX model is introduced to construct a Gaussian process autoregressive model, which uses the confidence interval of the prediction to describe the uncertainty, reduces the influence of model uncertainty on the prediction results, and improves the prediction rationality, accuracy, and efficiency of the joint angle prediction model.

## 2. Materials and Methods

### 2.1. Muscle Activation Dynamics

**Figure 1:**
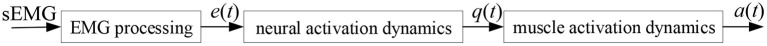
Muscle activation dynamics.

The sEMG signal is the sum of action potentials recruited to the muscle by surface electrodes and is used to reflect the activation level of the muscle. sEMG signals can be considered as a form of characterization of neuromotor control commands and are widely used to analyze musculoskeletal models. The feature extraction of the EMG signals by the existing studied models only considers the macroscopic characteristics of the EMG signals, without considering the microscopic physiological characteristics of muscle activation. In order to characterize the time-varying features of the sEMG signals, respond to micro-physiological properties, and reflect the relationship between EMG signals, neural activation and muscle activation, a muscle activation kinetic model was established to achieve feature extraction of the sEMG signals (29–31). The muscle activation kinetics is mainly expressed as the transformation process between EMG signals and muscle activation, as shown in Figure 1, where *a*(*t*) is the muscle activation, *e*(*t*) is the processed sEMG signal, *q*(*t*) the neural activation, and detailed in Li et al. (32).

### 2.2. Gaussian Process

Gaussian process (GP) is defined as a random process consisting of infinite high-dimensional random variables in a high-dimensional space, in which the joint distribution among any finite number of random variables is a Gaussian distribution. GP model can be derived from the weight-space view or the function-space view. Since each set of weights implies a specific function and the distribution of the weights implies the distribution of the function, the distribution of the GP can be obtained from the function-space view to obtain the equivalent of the weight-space view (33), which is the more commonly used derivation method for Gaussian process models.

Suppose the sample set D has *N* samples:

(1) $$\begin{array}{l} D=\left(\text{X},\text{Y}\right)=\left\{\left({\text{x}}_{i},y_{i}\right)|{\text{x}}_{i}\in {\mathbb{R}}^{d},y_{i}\in \mathbb{R},i=1,\ldots ,N\right\} \end{array}$$

where **x**_*i*_ denotes input vector,*y*_*i*_ denotes output vector.

A Gaussian process is completely specified by its mean function *m*(**x**) and covariance function *k*(**x**, **x′**) (34):

(2) $$\begin{array}{l} f\left(\text{x}\right)\sim GP\left(m\left(\text{x}\right),k\left(\text{x},{\text{x}}^{\prime }\right)\right) \end{array}$$

(3) $$\begin{array}{l} m\left(\text{x}\right)\;=\;𝔼\left[f\left(\text{x}\right)\right] \end{array}$$

(4) $$\begin{array}{l} k\left(\text{x},{\text{x}}^{\prime }\right)\;=\;𝔼\left[\left(f\left(\text{x}\right)-m\left(\text{x}\right)\right)\left(f\left({\text{x}}^{\prime }\right)-m\left(\text{x}\right)\right)\right] \end{array}$$

where the random variable function *f*(**x**) represents the distribution of *y*_*i*_ at **x**_*i*_, the mean function *m*(**x**) reflects the expected function value at input **x**. The covariance function models the dependence between the function values at different input points **x** and **x′**, which is often referred as the kernel function of a GP model.

In the Gaussian process regression, considering the following model:

(5) $$\begin{array}{l} \text{Y}\;=f\left(\text{X}\right)+\varepsilon \end{array}$$

where **X** denotes the input vector,**Y** denotes the observed vector with noise, the noise follows a Gaussian distribution $\varepsilon \sim N\left(0,\sigma^{2}\right)$, the random variable function *f*(**X**) follows a Gaussian distribution:

(6) $$\begin{array}{l} f\left(\text{X}\right)\sim N\left(\mu \left(\text{X}\right),\text{K}\left(\text{X},\text{X}\right)\right) \end{array}$$

Thus:

(7) $$\begin{array}{l} \text{Y}\sim N\left(\mu \left(\text{x}\right),\text{K}\left(\text{X},\text{X}\right)+\sigma^{2}I\right) \end{array}$$

For the prediction input $X^{\text{*}}\;\text{=}\;{\left(x_{1}^{*},\ldots ,x_{N}^{*}\right)}^{T}$, the joint distribution of the predicted values *f*(X^*^) and the training data output is:

(8) $$\begin{array}{l} \left(\begin{array}{c} \text{Y}\\ f\left({\text{X}}^{*}\right) \end{array}\right)\sim N\left(\left[\begin{array}{c} \mu \left(\text{X}\right)\\ \mu \left({\text{X}}^{*}\right) \end{array}\right],\left[\begin{array}{cc} \text{K}\left(\text{X},\text{X}\right)+\sigma^{2}\text{I} & \text{K}\left(\text{X},{\text{X}}^{*}\right)\\ \text{K}\left({\text{X}}^{*},\text{X}\right) & \text{K}\left({\text{X}}^{*},{\text{X}}^{*}\right) \end{array}\right]\right) \end{array}$$

where **K**(**X**, **X**) denotes the covariance matrix of the input signal, which is a symmetric semi-positive definite matrix of *N* × *N* order:

(9) $$\begin{array}{l} \text{K}\left(\text{X},\text{X}\right)\;=\;\left(\begin{array}{ccc} k\left({\text{x}}_{1},{\text{x}}_{1}\right) & \cdots & k\left({\text{x}}_{1},{\text{x}}_{N}\right)\\ \vdots & \ddots & \vdots \\ k\left({\text{x}}_{N},{\text{x}}_{1}\right) & \cdots & k\left({\text{x}}_{N},{\text{x}}_{N}\right) \end{array}\right) \end{array}$$

Knowing the joint high-dimensional distribution, the posterior distribution is obtained by finding the conditional probability *p*(*f*(X^*^)|**Y**, **X**, X^*^) according to Bayes' theorem:

(10) $$\begin{array}{l} f\left({\text{X}}^{*}\right)|{\text{X}}^{*}\sim N\left(\mu^{*},\Sigma^{*}\right) \end{array}$$

(11) $$\begin{array}{l} \mu^{*}\;\text{=}\;K(X^{*},X)[k{\left(X,X+\sigma^{2}I\right]}^{-1}(Y-\mu (X))+\mu (X^{*}) \end{array}$$

(12) $$\begin{array}{l} \Sigma^{*}=\text{K}\left({\text{X}}^{*},{\text{X}}^{*}\right)-\text{K}\left({\text{X}}^{*},\text{X}\right){\left[\text{K}\left(\text{X},\text{X}\right)+\sigma^{2}\text{I}\right]}^{-1}\text{K}\left(\text{X},{\text{X}}^{*}\right) \end{array}$$

The covariance matrix accounts for the major part of the posterior distribution of the Gaussian process, and the covariance matrix is a key component of the Gaussian process prediction. The kernel function is the main structure of the covariance matrix, so it is also a central part of the Gaussian process model. In the modeling and identification of dynamic systems, the dimensions of the inputs are relatively high, which makes the description of the mapping function complex. Considering the smoothness and continuity of dynamic systems, the squared exponential (SE) kernel function is often used in the modeling process (35), which is defined as:

(13) $$\begin{array}{l} k\left(\text{x},{\text{x}}^{\prime }\right)=\sigma_{f}^{2}\exp\left[-\frac{{\left(\text{x}-{\text{x}}^{\prime }\right)}^{T}\left(\text{x}-{\text{x}}^{\prime }\right)}{2\sigma_{l}^{2}}\right] \end{array}$$

where hyperparameter σ_*l*_ is the characteristic length scale, which determines the relative weights of the distances of the input variables. σ_*f*_ is the signal standard deviation, which reflects the magnitude of the function change.

The Gaussian process model is mainly determined by the kernel function and its hyperparameters, and its learning process is a process of training through the data to obtain the posterior probability distribution, which mainly includes the selection (or design) of the kernel function and the determination of the hyperparameters.

The kernel functions of Gaussian processes often contain unknown and indefinite hyperparameters, such as length scales, signal and noise variances, etc. These need to be inferred from the data, resulting in posterior distributions of the hyperparameters that are not easily obtained. Therefore, the full Bayesian derivation of hyperparameters is not commonly used in practical applications. The usual practice is to obtain point estimates of the hyperparameters by maximizing the log marginal likelihood.

Given a sample set D and the hyperparameter of the Gaussian process is θ, the marginal likelihood is as shown in Equation (14):

(14) $$\begin{array}{l} p\left(\text{Y}|\text{X},\theta \right)=\int p\left(\text{Y}|\text{X},f,\theta \right)p\left(f|\text{X},\theta \right)df \end{array}$$

The marginal likelihood is mainly a marginalization of the function. In the Gaussian process model, the prior *f*|**X**, ***θ*** of the model is a Gaussian distribution, i.e., $p\left(f|\text{X},\theta \right)=N\left(0,{\text{K}}_{\theta }\right)$. When the observed likelihood function *p*(**Y**|**X**, *f*, ***θ***) of the sample set is also Gaussian distributed, i.e., $p\left(\text{Y}|\text{X},f,\theta \right)=N\left(f,\sigma^{2}\text{I}\right)$, then *p*(**Y**|**X**, ***θ***) is also Gaussian distributed:

(15) $$\begin{array}{l} p\left(\text{Y}|\text{X},\theta \right)=\int N\left(0,{\text{K}}_{\text{Y}}\right)N\left(f,\sigma^{2}\text{I}\right)df=N\left(0,{\text{K}}_{\text{Y}}+\sigma^{2}\text{I}\right) \end{array}$$

According to Equation (15), the log marginal likelihood is obtained as:

(16) $$\begin{array}{l} \log p\left(\text{Y}|\text{X},\theta \right)=-\frac{1}{2}{\text{Y}}^{T}{{\text{K}}_{\text{Y}}}^{-1}\text{Y}-\frac{1}{2}\log|{\text{K}}_{\text{Y}}|-\frac{N}{2}\log2\pi \end{array}$$

where ${\text{K}}_{\text{Y}}\;=\;\text{K}\left(\text{X},\text{X}\right)+\sigma^{2}\text{I}$ is the output covariance matrix.

The maximum likelihood estimation combined with the conjugate gradient method is commonly used for the Gaussian process model to achieve the estimation of the hyperparameters of the model, and the computational complexity of this method is $O\left(N^{2}\right)$ for each hyperparameter, and the computational complexity is small. The hyperparameter estimates of the Gaussian process model are obtained by maximizing the log marginal likelihood function through a gradient ascent based optimization tool:

(17) $$\begin{array}{l} \begin{array}{c} \frac{\partial }{\partial \theta_{i}}\log p\left(\text{Y}|\text{X},\theta \right)=-\frac{1}{2}{\text{Y}}^{T}{{\text{K}}_{\text{Y}}}^{-1}\frac{\partial {\text{K}}_{\text{Y}}}{\partial \theta_{i}}{{\text{K}}_{\text{Y}}}^{-1}\text{Y}-\frac{1}{2}tr\left({{\text{K}}_{\text{Y}}}^{-1}\frac{\partial {\text{K}}_{\text{Y}}}{\partial \theta_{i}}\right)\\ \;=\;\frac{1}{2}tr\left(\left({\alpha \alpha }^{T}-{{\text{K}}_{\text{Y}}}^{-1}\right)\frac{\partial {\text{K}}_{\text{Y}}}{\partial \theta_{i}}\right) \end{array} \end{array}$$

where $\alpha \;=\;{\text{K}}_{\text{Y}}^{-1}\text{Y}$.

### 2.3. Non-parametric Model for Joint Angle Prediction Based on sEMG Signals

#### 2.3.1. Joint Angle Prediction Based on Gaussian Process Model

**Figure 2:**
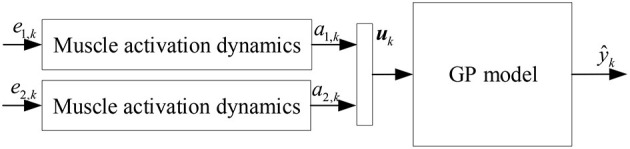
Joint angle prediction based on Gaussian process model.

The hamstrings and quadriceps are antagonistic muscles that together control the flexion and extension of the knee joint. The hamstrings are the muscles of the posterior thigh and consist mainly of the semitendinosus, semimembranosus and biceps femoris, while the quadriceps are the muscles of the anterior thigh and consist mainly of the vastus lateralis, vastus medialis, vastus intermedius and rectus femoris. In this paper, the sEMG signals of a pair of muscles in this antagonistic muscle group, the semimembranosus and the lateral femoris, were selected for the development of a non-parametric model for joint angle prediction. The physiological properties of muscle activation are combined with the GP model, and the squared exponential kernel is selected for subsequent modeling and analysis to establish a non-parametric model for joint angle prediction based on the GP model, as shown in Figure 2, where *k* denotes *k*th time step, *e*_1, *k*_ and *e*_2, *k*_ are the preprocessed sEMG signals of semimembranosus and lateral femoris muscles, respectively, which are then subjected to muscle activation dynamics to calculate the muscle activation *a*_1, *k*_ and *a*_2, *k*_. ***u***_*k*_ denotes the input of the GP model, $u_{k}={\left[a_{1,k},a_{2,k}\right]}^{T}$. The output of the Gaussian process model ŷ_*k*_ is the predicted value of the joint angle.

#### 2.3.2. Joint Angle Prediction Based on Gaussian Process Autoregressive Model

Since the neuromusculoskeletal system is a dynamic time-varying system, the joint angle prediction based on GP model only maps the input to the output distribution, which is a basic static system. Therefore, this paper further considers the time series of input and output signals to establish dynamic Gaussian process regression.

Non-linear autoregressive with eXogenous inputs (NARX) model is an effective method for solving non-linear sequence problems that accommodates non-linear discrete time processes and noisy models (36, 37). NARX is a dynamic recurrent network that predicts the current value of the system output by using a non-linear function f with previous inputs and outputs. The NARX model based on the actual measured values of the output is called One-step ahead (OSA) prediction:

(18) $$\begin{array}{l} y_{k}^{*}=f\left(u_{k},u_{k-1}\ldots ,u_{k-n_{u}},y_{k-1},y_{k-2},\ldots ,y_{k-n_{y}}\right) \end{array}$$

where *f*(·) is the non-linear function between the input *u*_*k*_ and the estimated value $y_{k}^{*}$ and *y*_*k*_ denotes measured value of model output, *k* represents *k*th time step, *n*_*u*_ and *n*_*y*_ are the maximum lags for model input and output, respectively.

**Figure 3:**
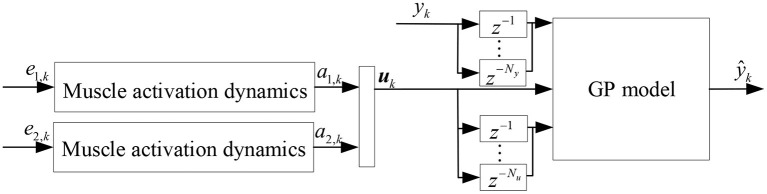
Joint angle prediction based on Gaussian process autoregressive model.

The joint angle signal can be easily collected by inertial measurement unit (IMU), etc., and the joint angle prediction system can be established by EMG signals to achieve further advance prediction of joint angle. Since the high accuracy of joint angle prediction is required in practical applications, this paper improves the joint angle prediction method based on GP model by using the NARX model and muscle activation dynamics, which establishes a Gaussian dynamic model with NARX structure, i.e., Gaussian process autoregressive model, for joint angle OSA prediction, as shown in Figure 3, where *y*_*k*_ denotes measured value of joint angle, ŷ_*k*_ denotes the joint angle prediction of Gaussian process autoregressive model, *N*_*y*_ and *N*_*y*_ are the maximum lags for model input and output, respectively.

## 3. Experiments and Results

### 3.1. Datasets

In this paper, publicly available datasets (dataset 1 and dataset 2) were cited to validate and analyze the proposed knee joint angle prediction model.

#### 3.1.1. Healthy Subjects Dataset

Dataset 1 contains gait data from 10 healthy subjects in running condition, and Dataset 1 is published in https://simtk.org/projects/nmbl_running. The dataset measured knee moment signals, EMG signals and motion data of 10 healthy subjects running on a treadmill at four speeds (2.0, 3.0, 4.0, and 5.0 m/s). Subjects were all male (age: 29 ± 5 years; height: 1.77 ± 0.04 m; weight: 70.9 ± 7.0 kg), all provided informed consent, and each subject was experienced in long-distance running, at least 50 km per week. Fifty-four reflective markers were placed on each subject, and the trajectory of the markers was recorded using eight Vicon MX40+ cameras with a data acquisition frequency of 100 Hz. Ground reaction forces and moments were acquired using a Bertec Corporation treadmill with a sampling frequency of 1,000 Hz. Motion data and ground reaction forces were preprocessed with 4th order zero-phase hysteresis Butterworth low-pass filtering (cutoff frequency 15 Hz) and critical damping low-pass filtering (cutoff frequency 15 Hz), respectively. The Delsys Bangoli system was used to collect EMG signals. A total of 11 muscles including the gluteus maximus, biceps femoris long head, medial femoris, lateral femoris, semimembranosus, and tibialis anterior muscles were collected. Hamner et al. gave a complete description of the dataset (38).

#### 3.1.2. Hemiparetic Subject Dataset

Joint angle prediction is mainly applied to the development of rehabilitation equipment and rehabilitation training, so a gait dataset containing a male patient with high-functioning right hemiparesis, which is publicly available at https://simtk.org/projects/emgdrivenmodel, was also selected for further testing and analysis of the knee angle prediction model proposed in this paper. The subject was 79 years old, height 1.7 m, mass 80.5 kg, with a LE Fugl-Meyer motor assessment score of 32/34 and right-sided hemiparesis. All experimental procedures were approved by the University of Florida Health Sciences Center Institutional Review Board (IRB-01), and the subject signed a written informed consent prior to participation in the experiment. The dataset collected gait data from subjects walking on a split-belt instrumented treadmill (Bertec Corp., Columbus, OH) at five different speeds (0.4, 0.5, 0.6, 0.7, and 0.8 m/s), with over 50 gait cycles collected for each speed. Motion capture in the experiments was mainly performed by an optical motion capture system (Vicon Corp., Oxford, UK) and ground reaction force detection was measured using the treadmill with sampling frequency of 100 and 1,000 Hz, respectively. The EMG signal acquisition was performed using Motion Lab Systems with a sampling frequency of 1,000 Hz. Ground response and marker motion data was filtered using a fourth-order zero-phase lag Butterworth filter with a cutoff frequency of 7 divided by the gait period. EMG signal data were collected for 16 muscle groups of the lower extremity, including the anterior tibialis, semimembranosus, long head of the biceps femoris, medial femur, and lateral femur. A full description of this dataset is provided by Meyer et al. (39).

#### 3.1.3. Pre-processing and Feature Extraction Results

The sEMG signals of dataset 1 and dataset 2 are preprocessed. Raw sEMG signals was firstly filtered using Butterworth zero phase shift bandpass filter (4th order, cutoff frequency 40 Hz) to eliminate low-frequency noise, then full-wave rectified and low-pass filtered (4th order, cutoff frequency = 3.5/step period), and finally sEMG data from each muscle were normalized to the maximum value over all trials to obtain the processed sEMG signals *e*(*t*).

**Figure 4:**
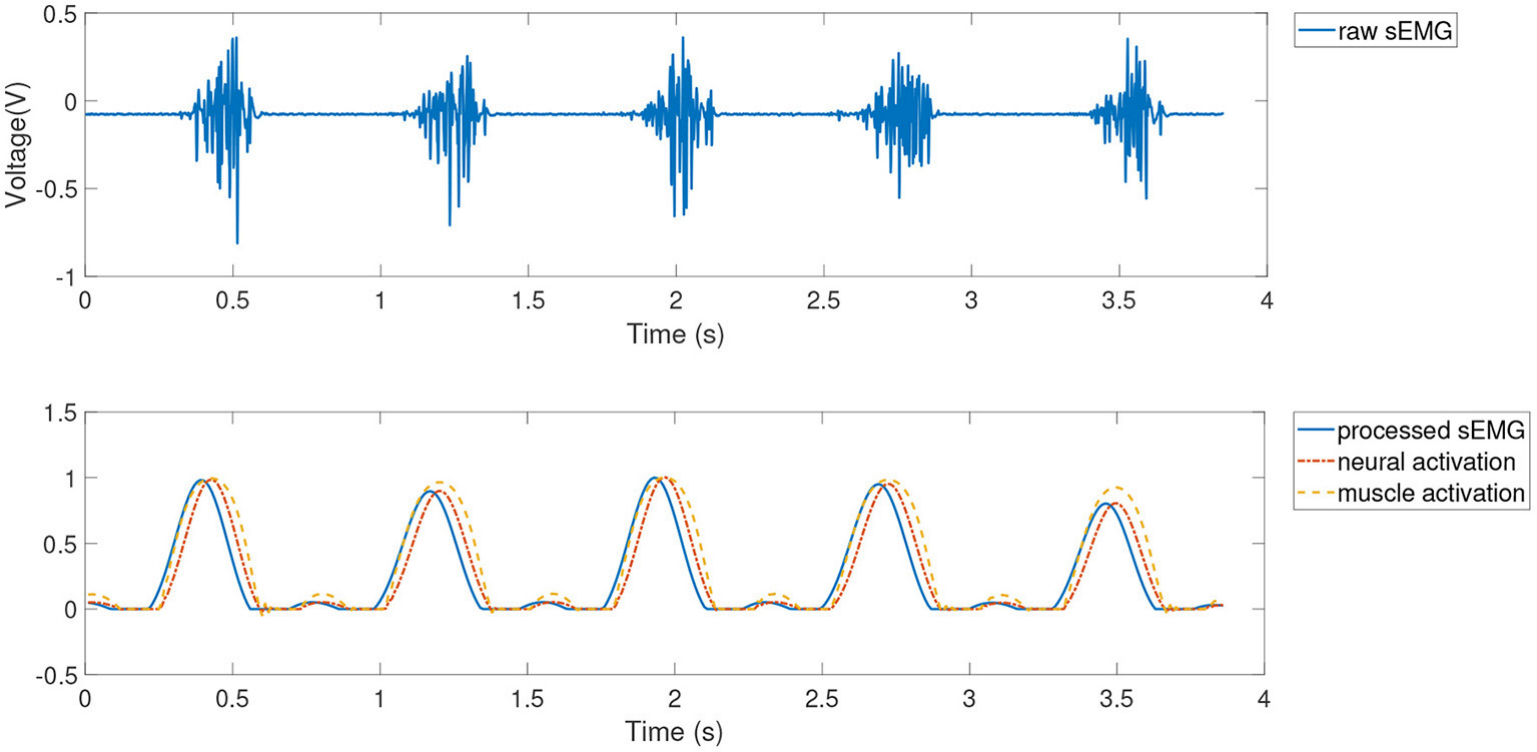
Feature extraction of the lateral femoral.

**Figure 5:**
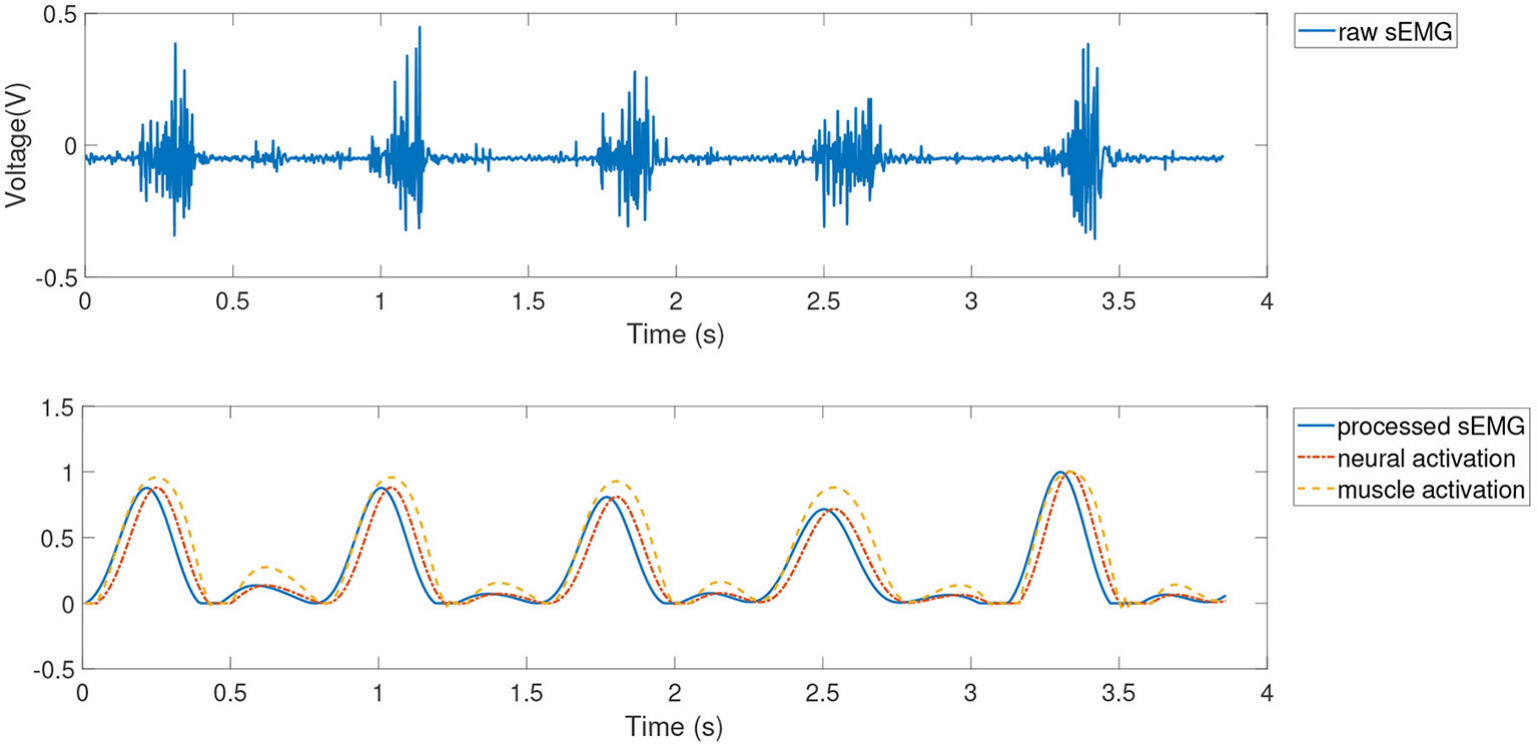
Feature extraction of semimembranosus.

Feature extraction was then further performed using the muscle activation kinetic model in section 2.1. The results of pre-processing and feature extraction for dataset 1 (subject 1, 2 m/s) for the lateral femoral and semimembranosus muscles are shown in Figures 4, 5.

### 3.2. Data Allocation Strategy

To study the effect of different speeds on joint angle prediction, joint angle prediction results were analyzed for two datasets of healthy subjects and hemiplegic subject in three conditions: speed-dependent, multi-speed and speed-independent. The normalized root mean square error (NRMSE) and correlation coefficient (CC) were used as evaluation indicators of the prediction performance of the joint angle, as shown in Equations (19) and (20).

(19) $$\begin{array}{l} NRMSE=\frac{1}{y_{real}^{\max}}\sqrt{\frac{1}{num}\sum_{i=1}^{num}{\left(y_{est}-y_{real}\right)}^{2}} \end{array}$$

(20) $$\begin{array}{l} CC=\frac{cov\left(y_{est},y_{real}\right)}{\sigma_{y_{est}}\cdot \sigma_{y_{real}}} \end{array}$$

where num denotes the number of samples tested, *y*_*est*_ denotes the predicted value of joint angle, *y*_*real*_ denotes the actual value of joint angle, and $y_{real}^{\max}$ denotes the maximum magnitude of the actual joint angle value.

One-way analysis of variance (ANOVA) was conducted to assess the statistical difference of estimation errors obtained by different models (40). The level of statistical significance was set to *p* < 0.05.

Dataset 1 contained 10 subjects, each speed containing five gait cycles, and three conditions were analyzed for each subject. speed-dependent took the first three cycles of each speed as a training set, and the last two cycles at the corresponding speed as test set; multi-speed took the first three cycles of each speed together as a training set, and the last two cycles of each speed separately as a test set; the speed-independent took the last two cycles of one speed in turn as test set, and the first three cycles of each unselected speed together as training set.

Dataset 2 contained the left and right leg gait data of a subject with right-sided hemiplegia, and the left and right leg gait data were analyzed for three conditions. speed-dependent tested the angle prediction results of each speed, using 10 gait cycles of a single speed as the training set and another 10 cycles of the same speed as the test set; multi-speed used data from 5 m/s, each with 10 gait cycles, for a total of 50 gait cycles as the training set, and 10 gait cycles for each speed as the test set; speed-independent test took 10 gait cycles of one speed in turn as the test set, and 10 gait cycles of each of the other unselected speeds together as the training set.

Before performing the joint angle prediction based on Gaussian process model and Gaussian process autoregressive model, the Gaussian process model needs to be trained offline for different datasets according to the data allocation strategy. In this paper, the dataset was trained and tested on the MATLAB platform, and the Gaussian process regression model was trained offline using the “fitrgp” function. The method of estimating the model parameters was set to “exact,” and the point estimates of the hyperparameters were obtained by maximizing the log marginal likelihood, and the kernel function was set to the squared exponential kernel function. The input signal for offline training of joint angle prediction based on Gaussian process model was the muscle activation of lateral femoral and semimembranosus muscles, and the output was the normalized joint angle signal. Given that muscle dynamics is a second-order model, the joint angle prediction based on the Gaussian process autoregressive model is set to second order, which lead to the number of the maximum lags for model input and output be 2, i.e., *n*_*u*_ = *n*_*y*_ = 2. Therefore, the output signal of offline training was the joint angle signal, and the input was the muscle activation. The model was trained offline and joint angle prediction was performed according to the data allocation strategy for different datasets under different data allocation strategies, respectively.

### 3.3. Estimation Results

#### 3.3.1. Joint Angle Prediction Results for Dataset 1

**Figure 6:**
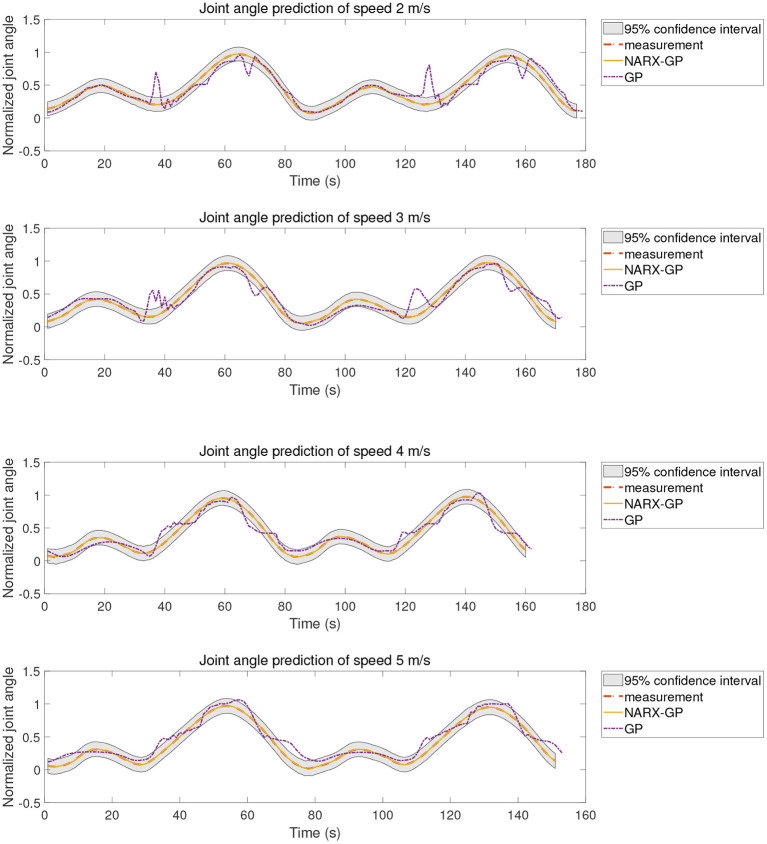
Joint angle prediction under speed-dependent of subject 9.

**Figure 7:**
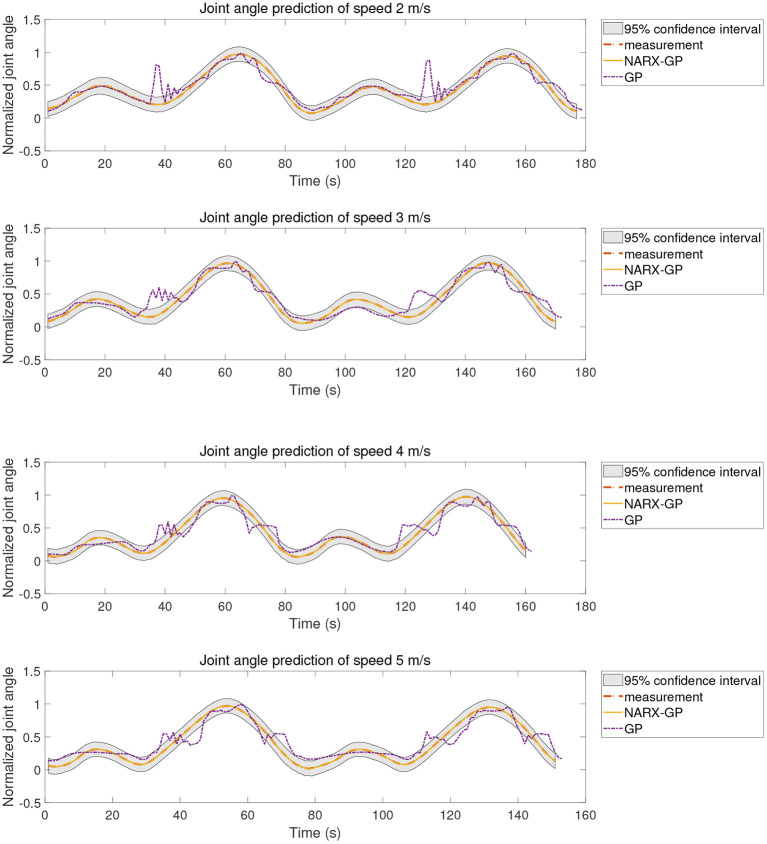
Joint angle prediction under multi-speed of subject 9.

**Figure 8:**
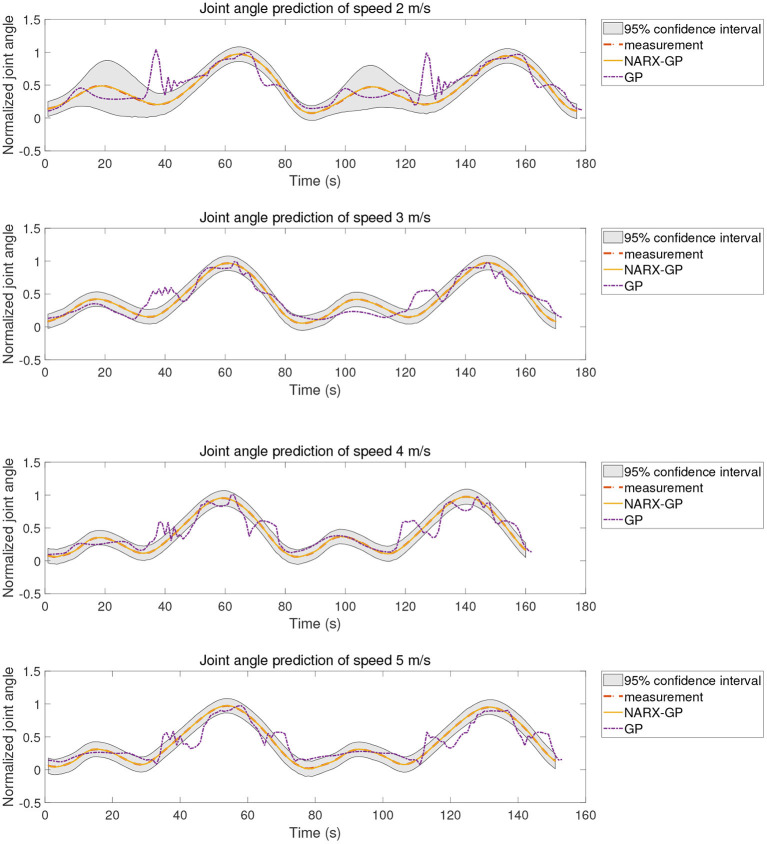
Joint angle prediction under speed-independent of subject 9.

The proposed method was tested using the data in dataset 1. Subjects were tested for knee angle prediction in three cases, speed-dependent, multi-speed, and speed-independent, according to the data allocation strategy, and the test results for subject 9 knee angle prediction were shown in Figures 6–8, where “NARX-GP” was the joint angle prediction based on the Gaussian process autoregressive model, “GP” was the joint angle prediction based on the Gaussian process model, and “measurement” was the actual measurement of joint angle. The gray shading was the 95% confidence interval (μ±2σ) for the prediction of the joint angle based on the Gaussian process autoregressive model to describe the uncertainty. From Figures 6–8, it can be concluded that the direct joint angle prediction by Gaussian process model cannot describe the relationship between sEMG signal and joint angle well, and the knee joint angle prediction results have a large error. Establishing a Gaussian process autoregressive model for OSA prediction of joint angle can significantly improve the prediction accuracy and can approximate the actual joint angle signal. OSA prediction incorporates the actual values of the previous moments of output into the model structure with high prediction accuracy, and is suitable for scenarios where the actual measurements of the output are easy to collect and where high prediction accuracy is required. The joint angle signal can be easily collected by inertial measurement unit (IMU), etc., and the joint angle prediction system can be established by EMG signals to achieve further advance prediction of joint angle.

**Figure 9:**
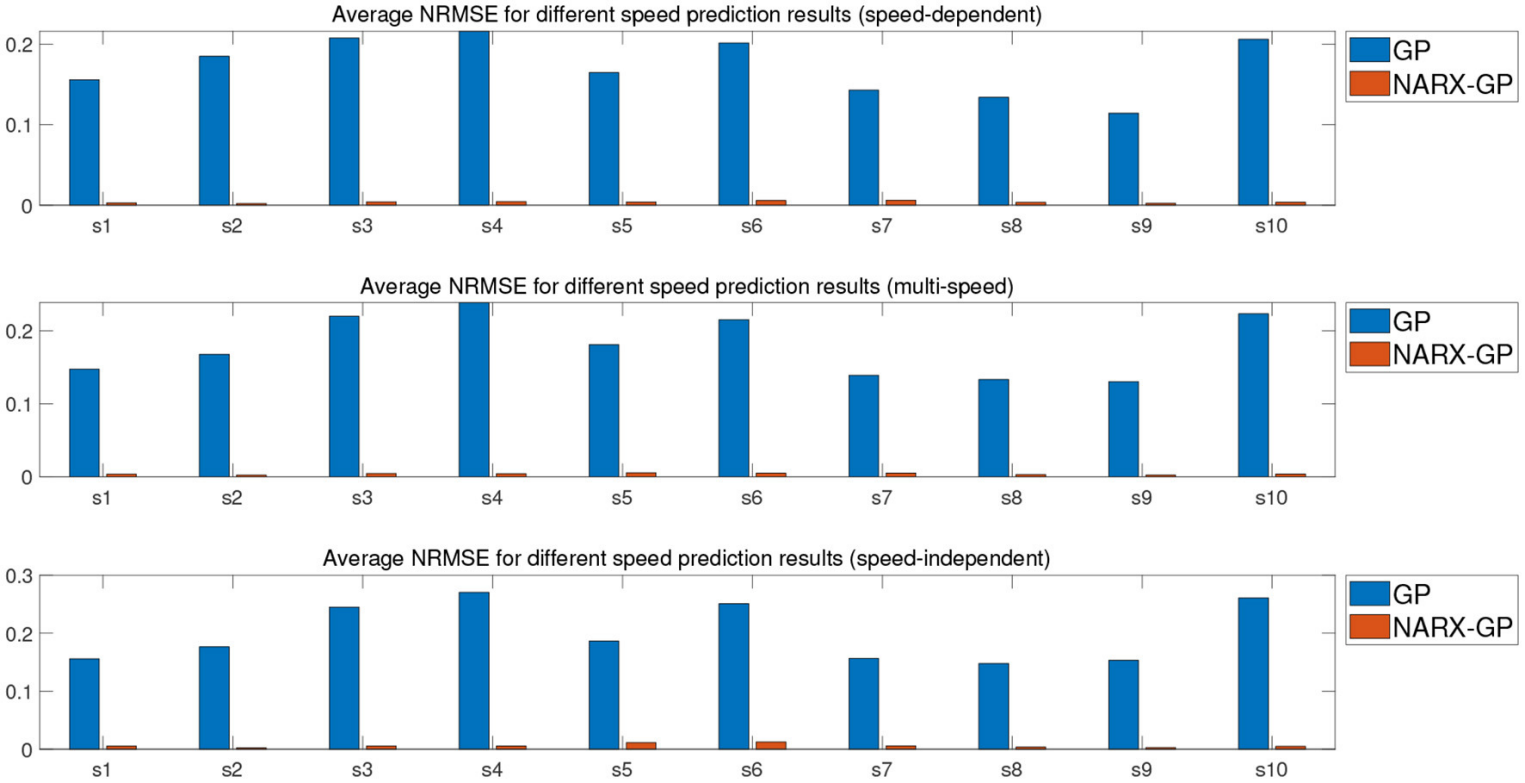
Average NRMSE for prediction of joint angle under different speed.

**Figure 10:**
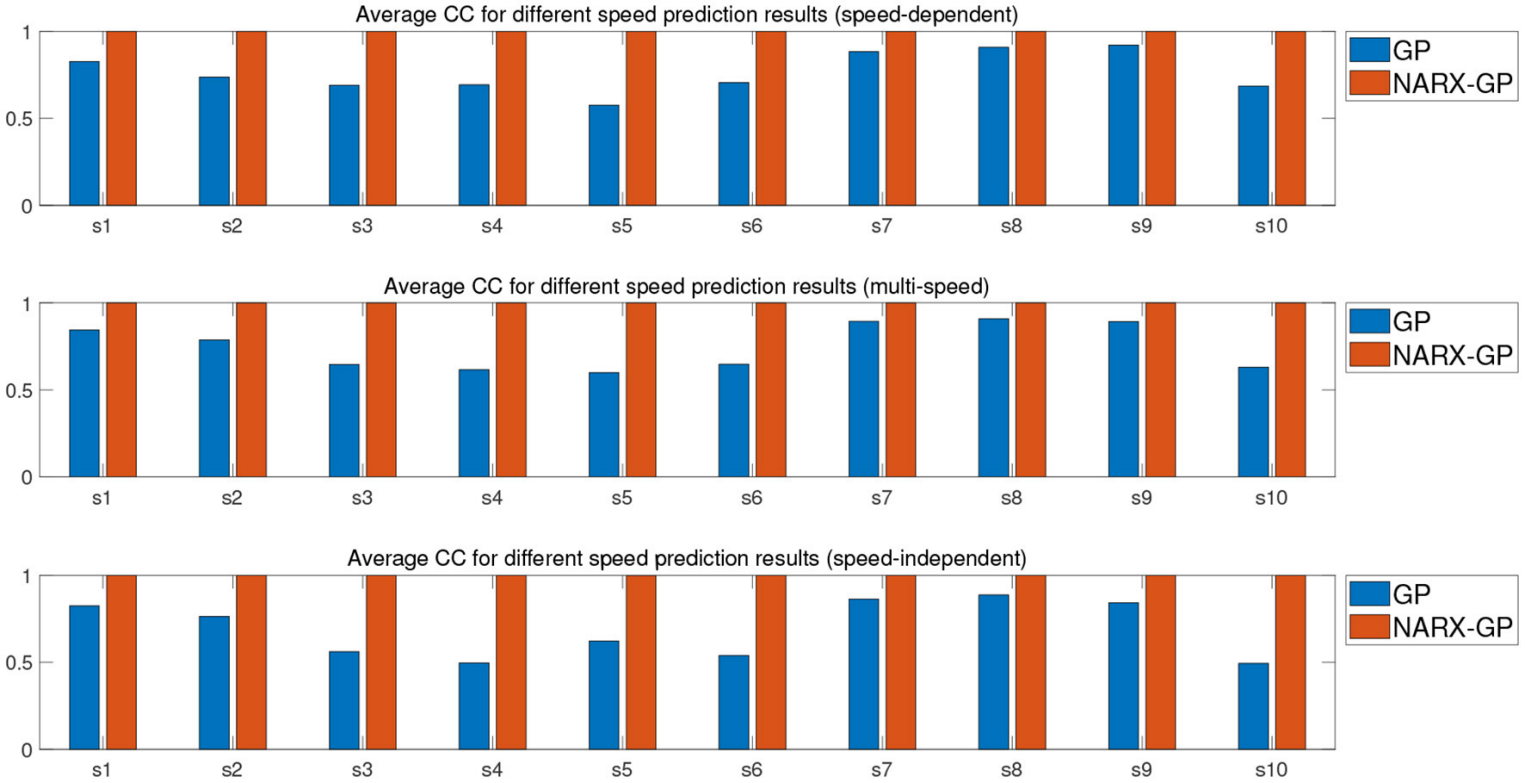
Average CC for prediction of joint angle under different speed.

Further error assessment and statistical analysis of the prediction results were performed, and the mean NRMSE and CC between the predicted and actual measurements for different velocity joint angles of the subjects are shown in Figures 9, 10. It can be seen from the figures that the prediction results of the NARX-GP model were significantly better than those of the GP model, the NRMSE between the prediction results of the NARX-GP model for knee joint angle and the actual knee joint angle was small and significantly smaller than that of the GP model, and the strong correlation between the prediction results of the NARX-GP model and the actual values of the joint angle with a higher correlation coefficient than that of the GP model.

The means and standard deviations of NRMSE and CC between predicted and actual values of joint angles for all subjects in speed-dependent, multi-speed and speed-independent conditions are shown in Tables 1, 2. From Tables 1, 2, it can be seen that the predictions of the NARX-GP model were highly correlated, and the NRMSE of the predictions was significantly lower than that of the GP model. The mean NRMSE was further calculated as 0.0039 ± 0.019, 0.0038 ± 0.0019, and 0.0059 ± 0.0061 for all subjects in the NARX-GP model in the load-dependent, multi-load and load-independent conditions, 0.1728 ± 0.0543, 0.1795 ± 0.0563, and 0.2003 ± 0.0659 for the GP model. ANOVA was used to evaluate NRMSE of NARX-GP and GP model predictions, showing significant differences (*p* < 0.05, *p* < 0.05, and *p* < 0.05). The NARX-GP model prediction errors were smallest for all three scenarios, and slightly larger for load-independent. The joint angle prediction results of the GP model for all three scenarios were optimal for speed-dependent, with the smallest NRMSE, followed by multi-speed, and worst for speed-independent. The variability of joint motion at different speeds affected the prediction results of the model, and the experimental results also demonstrated that the speed-dependent results were optimal and the speed-independent results were the worst. The average NRMSE of NARX-GP model prediction results for all scenarios was 0.0045 ± 0.0040, which was better than the GP model results (0.1842 ± 0.0602), with a significant difference (*p* < 0.05).

**Table 1:** NRMSE between the estimated joint torque of different models and the measurements (“true” values) of all subjects (mean ± std).

		**2 m/s**	**3 m/s**	**4 m/s**	**5 m/s**
Speed-dependent	GP	0.1788 ± 0.0756	0.1802 ± 0.0475	0.1604 ± 0.0483	0.1718 ± 0.0356
	NARX-GP	0.0046 ± 0.0016	0.0039 ± 0.0023	0.0031 ± 0.0023	0.0041 ± 0.0021
Multi-speed	GP	0.1850 ± 0.0735	0.1745 ± 0.0489	0.1712 ± 0.0457	0.1874 ± 0.0510
	NARX-GP	0.0052 ± 0.0028	0.0033 ± 0.0010	0.0030 ± 0.0011	0.0035 ± 0.0011
Speed-independent	GP	0.2075 ± 0.0697	0.1852 ± 0.0560	0.1910 ± 0.0563	0.2174 ± 0.0746
	NARX-GP	0.0086 ± 0.0086	0.0039 ± 0.0014	0.0046 ± 0.0030	0.0066 ± 0.0072

**Table 2:** CC between the predicted joint angle of different models and the measurements (“true” values) of all subjects (mean ± std).

		**2 m/s**	**3 m/s**	**4 m/s**	**5 m/s**
Speed-dependent	GP	0.6276 ± 0.4151	0.7819 ± 0.1202	0.8241 ± 0.1120	0.8170 ± 0.1080
	NARX-GP	0.9999 ± 0.0001	0.9999 ± 0.0002	0.9999 ± 0.00004	0.9999 ± 0.0001
Multi-speed	GP	0.6381 ± 0.3708	0.7744 ± 0.1573	0.8026 ± 0.1141	0.7710 ± 0.1591
	NARX-GP	0.9998 ± 0.0002	0.9999 ± 0.00003	0.9999 ± 0.00003	0.9999 ± 0.0001
Speed-independent	GP	0.5970 ± 0.3333	0.7387 ± 0.2172	0.7493 ± 0.1540	0.6733 ± 0.2494
	NARX-GP	0.9991 ± 0.0020	0.9999 ± 0.00006	0.9998 ± 0.0002	0.9996 ± 0.0009

#### 3.3.2. Joint Angle Prediction Results for Dataset 2

**Figure 11:**
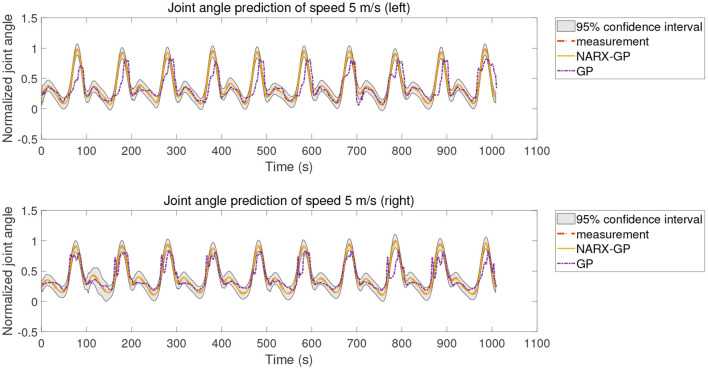
Joint angle prediction of 5 m/s (speed-dependent).

**Figure 12:**
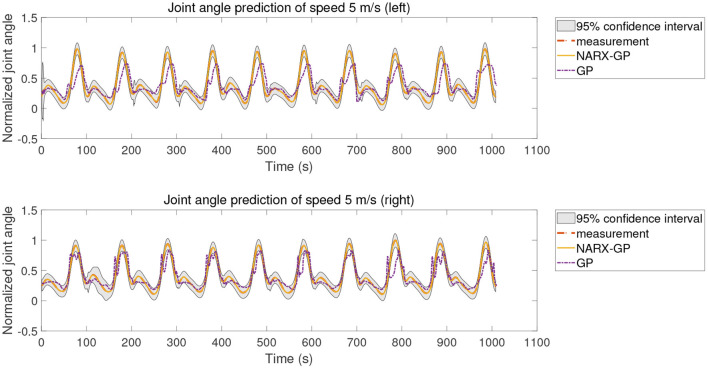
Joint angle prediction of 5 m/s (multi-speed).

**Figure 13:**
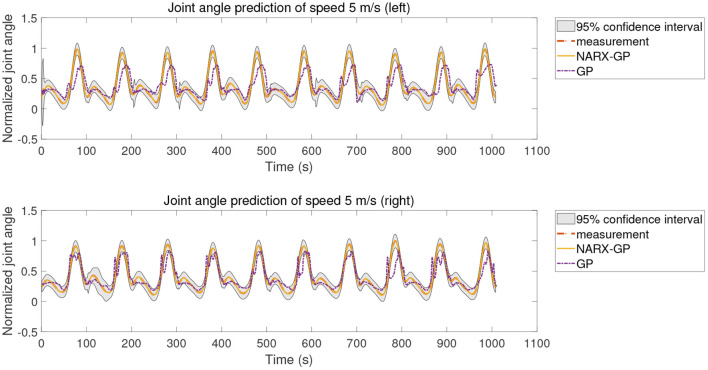
Joint angle prediction of 5 m/s (speed-independent).

The proposed method was further tested using the data in dataset 2 to validate the accuracy of the proposed method for predicting the knee joint angle of patients. To reduce the influence of different muscle selections on the prediction results, the sEMG signals of the same pair of muscles, semimembranosus and lateral femoris, were selected for testing in dataset 2 as in dataset 1 and used to build a non-parametric model for joint angle prediction. Dataset 2 included data from the left and right legs of patients with different speeds, so the joint angle prediction results of subjects in three cases of speed-dependent, multi-speed and speed-independent were tested separately for the left and right legs according to the data allocation strategy, and the test results of joint angle prediction for speed of 5 m/s are shown in Figures 11–13, where “NARX-GP” was the joint angle prediction based on the Gaussian process autoregressive model, “GP” was the joint angle prediction based on the Gaussian process model, and “measurement” was the actual measurement of joint angle. The gray shading was the 95% confidence interval for the prediction of the joint angle based on the Gaussian process autoregressive model to describe the uncertainty. From Figures 11–13, it can be concluded that the patient's knee joint NARX-GP model has better angle prediction than the GP model and can achieve good prediction results, and the joint angle prediction can approximate the actual joint angle for both the healthy side and the affected side (right side) of the patient.

**Figure 14:**
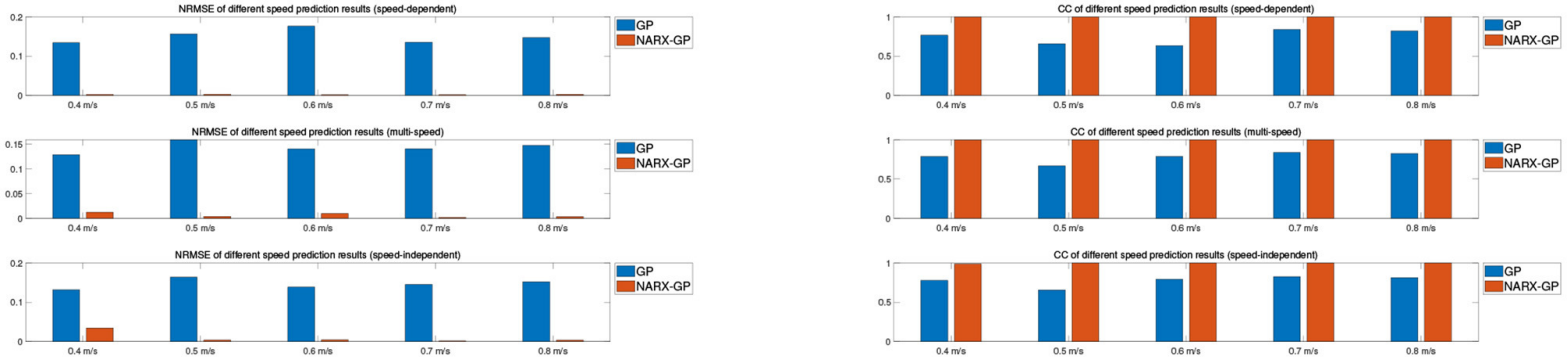
Angle prediction results of NARX-GP model and GP model for hemiplegic subject dataset.

**Figure 15:**
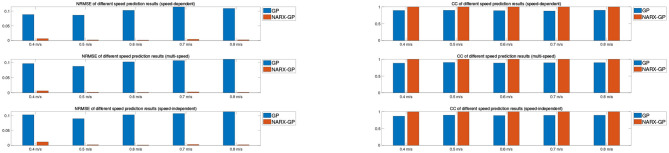
Angle prediction results of NARX-GP model and GP model for hemiplegic subject dataset.

The error assessment and statistical analysis of the prediction results of the NARX-GP model and the GP model for the hemiplegic subject dataset, the NRMSE and CC between the predicted and actual values of joint angles at different speeds are shown in Figures 14, 15. The errors between the predicted results and the actual values of joint angles for both the left and right leg NARX-GP models were small, highly correlated, and significantly better than the GP model.

Further evaluation and analysis of variance of the prediction results for the left and right legs of the dataset showed that the mean NRMSE of the prediction results of the NARX-GP model for the left and right legs were 0.0063 ± 0.0081 and 0.0032 ± 0.0028, respectively, which were significantly better than those of the GP model (0.1466 ± 0.0127 and 0.1012 ± 0.0092), with significant differences (*p* < 0.05 and *p* < 0.05). Joint angle prediction using the NARX-GP model for both the healthy and affected side of the patient was able to have high accuracy with no significant difference (*p* = 0.1919>0.05). the NRMSE of the NARX-GP model prediction results for all scenarios was 0.0047 ± 0.0063 on average, which was better than the GP model results (0.1239 ± 0.0253) with a significant difference (*p* < 0.05).

## 4. Discussion and Conclusion

The EMG signal contains abundant motion information, which is ahead of the actual joint motion, and is often used as a control signal to predict joint motion. Therefore, EMG signals are widely used in applied scientific research related to the development of intelligent rehabilitation technologies and devices. EMG signal based modeling of the neuromusculoskeletal system, as an important component of joint motion prediction, has become a hot topic of research as it is important to help the development of rehabilitation techniques and equipment for patients with sports injuries. There is uncertainty in the neuromusculoskeletal system, and in order for the model to provide a description of the uncertainty, this paper proposes to model the uncertainty using a Gaussian autoregressive model. The muscle activation dynamics model was first introduced into the Gaussian process model to establish a joint angle prediction model based on Gaussian process. Due to the high requirement for joint angle prediction accuracy in practical applications and the fact that the neuromusculoskeletal system is a dynamic non-linear system, the NARX model was introduced into the Gaussian process model to establish a Gaussian autoregressive model to achieve OSA prediction of knee joint angle. The results of different test scenarios on the healthy subjects and hemiplegic subject datasets showed that the designed Gaussian autoregressive model had significantly better prediction accuracy than the Gaussian process model, and there was no significant difference in the prediction accuracy between the affected and healthy sides of the hemiplegic subject, both of which were able to achieve more accurate prediction results for knee angles and could provide uncertainty information.

In this paper, a non-parametric model for knee joint angle prediction was developed from a predictive value-based NARX model approach by mixing a muscle activation kinetic model with a data-driven model. The proposed modeling approach was validated with a publicly available dataset. The proposed method utilizes only the EMG signals of a pair of antagonistic muscles, reducing the cost of EMG signal detection and the complexity of the model. However, there are still some shortcomings in this paper and there are many problems that have not yet been studied with some need for improvement. In this paper, the performance of only one pair of antagonist muscles in the hamstrings and quadriceps was tested, and the effect of sEMG signals from other muscles in the hamstrings and quadriceps as input on the accuracy of knee joint angle prediction can be further tested. In addition, although the knee joint is used as the research object for the study and validation of the model in this paper, the proposed joint angle prediction method is not limited to the knee joint angle prediction. In the subsequent research, the proposed method can be applied to the angle prediction of other joints for relevant testing and validation.

## Data Availability Statement

The original contributions presented in the study are included in the article/supplementary material, further inquiries can be directed to the corresponding author/s.

## Author Contributions

Conception of the study was conducted by JL, ZS, and WC. FZ processed the data. XC analyzed and interpreted the data with input and support from XC and JL. WC drafted the manuscript. YL, ZS, and WC revised the manuscript critically for important intellectual content. All authors read and approved the manuscript.

## Conflict of Interest

The authors declare that the research was conducted in the absence of any commercial or financial relationships that could be construed as a potential conflict of interest.
